# Dual-Band Passive Beam Steering Antenna Technologies for Satellite Communication and Modern Wireless Systems: A Review

**DOI:** 10.3390/s24186144

**Published:** 2024-09-23

**Authors:** Maira I. Nabeel, Khushboo Singh, Muhammad U. Afzal, Dushmantha N. Thalakotuna, Karu P. Esselle

**Affiliations:** School of Electrical and Data Engineering, University of Technology Sydney, Sydney, NSW 2007, Australia; khushboo.singh@uts.edu.au (K.S.); muhammad.afzal@uts.edu.au (M.U.A.); dushmantha.thalakotuna@uts.edu.au (D.N.T.); karu.esselle@uts.edu.au (K.P.E.)

**Keywords:** beam steering, metasurface, dual-band, SATCOM, LEO, antenna, near field, unit cell, phase-gradient metasurface, phase shifting surface, COTM

## Abstract

Efficient beam steerable high-gain antennas enable high-speed data rates over long-distance networks, including wireless backhaul, satellite communications (SATCOM), and SATCOM On-the-Move. These characteristics are essential for advancing contemporary wireless communication networks, particularly within 5G and beyond. Various beam steering solutions have been proposed in the literature, with passive beam steering mechanisms employing planar metasurfaces emerging as cost-effective, power-efficient, and compact options. These attributes make them well-suited for use in confined spaces, large-scale production and widespread distribution to meet the demands of the mass market. Utilizing a dual-band antenna terminal setup is often advantageous for full duplex communication in wireless systems. Therefore, this article presents a comprehensive review of the dual-band beam steering techniques for enabling full-duplex communication in modern wireless systems, highlighting their design methodologies, scanning mechanisms, physical characteristics, and constraints. Despite the advantages of planar metasurface-based beam steering solutions, the literature on dual-band beam steering antennas supporting full duplex communication is limited. This review article identifies research gaps and outlines future directions for developing economically feasible passive dual-band beam steering solutions for mass deployment.

## 1. Introduction

**Figure 1:**
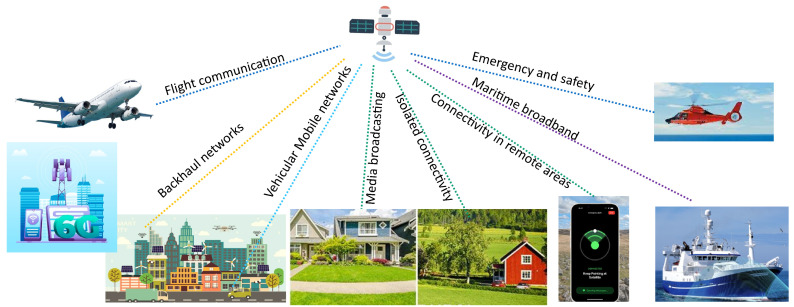
The modern wireless communication landscape.

With an ever-increasing demand for seamless global connectivity, the satellite terminal market has experienced exponential growth [1]. This surge is also driven by the rapid advancement of technologies such as the Internet of Things (IoT), communication on the move (COTM), in-flight internet connectivity, maritime and offshore connectivity, and efforts to bridge the connectivity gap in underserved areas. The integration of IoT in the aviation industry is poised to revolutionize operations, using satellite communication (SATCOM) to transmit and receive real-time data between IoT systems and components. From providing Internet access in remote regions to facilitating emergency services in critical situations, SATCOM has the potential to benefit many sectors. According to reports from the International Telecommunication Union (ITU), ≈67% of the world’s population uses the Internet, of which 59% of the web traffic worldwide is through mobile devices [2,3]. In such scenarios, satellite communication is the most appropriate solution for mobile connectivity when terrain, line of sight, or distance restrict connectivity via standard terrestrial networks. Wireless telecommunication providers intend to use more and more SATCOM nodes to extend the coverage provided by terrestrial networks in sparsely populated regions [4,5,6]. The necessity for ubiquitous connectivity for some of the platforms in the present-day communication landscape is clearly illustrated in Figure 1.

Some of the typical commercial communication satellite systems include Iridium-NEXT, LeoSat, OneWeb, Starlink, O3B, and Kuiper [7,8]. Most satellite communication networks utilize low-earth orbit (LEO) and medium-earth orbit (MEO) satellites, which are preferred over geostationary (GEO) satellites due to their proximity to Earth. This proximity results in lower power consumption and lower latency [9,10,11]. These LEO satellites utilize the Ku/Ka bands for full-duplex communication. Since LEO satellites are non-stationary, beam steering antennas maintain connectivity with the satellite by constantly switching beams from one satellite to another. Moreover, medium-to-high gain antennas are required since they can close the link budget with lesser transmission power, reducing the power burden on the radio frequency (RF) system [12].

## 2. Beam Steering Antenna Technologies

Based on the steering principles involved, beam steering antennas can be broadly classified as mechanical or electrical. [13]. Electrical antenna systems can be further classified as phased arrays or beamforming antennas, including digital, hybrid and analog beamforming [13,14].

In mechanical beam steering, the antenna system is physically rotated with the help of electric motors to direct the beam in any direction. High-gain antennas such as reflector dishes or arrays of horns are typical examples of mechanically steered antennas [5,15]. Various mechanical steering antennas for SATCOM On-The-Move (SOTM) are commercially available [16]. Although mechanical parabolic dish-based steering methods maintain antenna gain and offer flexible steering ranges, such systems are undesirable in certain applications due to antenna weight and size, weather effects, and steering speeds. Some of the mechanical steering systems are bulky and expensive. Power requirements are also increased when the complete system has to be rotated. Such designs may not be feasible when the whole system has to be incorporated into a smaller space to reduce the payload. Likewise, for mobile platforms such as cars, trains, or aircraft, integrating a mechanically rotating system proves impractical. In these scenarios, where aerodynamic considerations are paramount, compact and planar antenna systems reign supreme over their bulkier, mechanically rotated counterparts.

The other technique uses phased arrays, an electrical beam steering technique that can provide adaptive beam scanning and beam shaping capabilities [17]. The currently available active RF-based electrical steering antennas, including [18,19], utilize electrical beam steering by incorporating active metasurfaces for full-duplex shared aperture antenna operation. Several other antenna systems are available for SOTM and COTM [20,21]. Although electrical beam steering systems provide faster scanning speed and compact antenna designs, they require complex feed networks due to the distributed amplifiers and phase shifters in the feed network compared to passive systems with mechanical means to steer the beam.

Lately, there has been a notable increase in research interest in metasurface-based beam steering techniques [22,23,24,25]. These techniques are particularly attractive because they offer cost-effective solutions for planar antennas well-suited for large-scale deployment. Metasurface-based beam steering techniques function similarly to phased arrays, but instead of using RF phase shifters, they employ phase delay unit cells [26,27] or phase rotation unit cells [28]. This innovation enables flexible beam manipulation while reducing the complexity associated with conventional phased-array implementations. Since the passive metasurface does not include any active circuits to introduce phase shifts or control the direction of the beam, it does not require any RF power for its operation. Additionally, because the entire surface is lightweight, low-power DC motors can be used to rotate it. Compared to some reported phased arrays [29,30], the antenna’s gain is higher in [31,32,33], which means less RF input power is needed to achieve the same effective isotropically radiated power (EIRP) as reported in [29,30].

**Figure 2:**
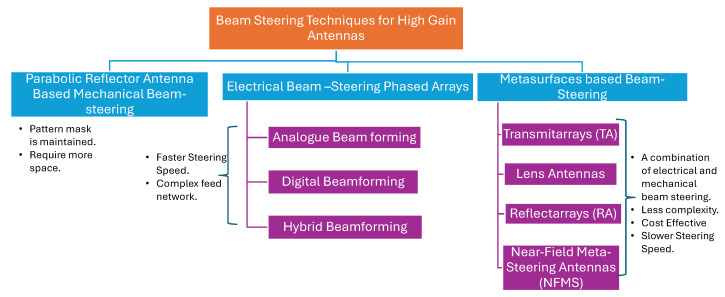
Classification of beam steering techniques.

The transmitarrays (TA), refelctarrays (RAs), and near-field meta-steering (NFMS) antennas reported in the literature, have aperture efficiency values ranging from 15% to 63% [31,32,33]. Therefore we calculated the gain-to-noise temperature, G/T, assuming an aperture efficiency of 34%, which is the median value. For an antenna with aperture efficiency 34% and the same physical area as the one reported in [29], the calculated gain (G) is 36.14 dB. Assuming a similar receiving system is connected at the back end of the antenna, the calculated noise temperature (T) for a complete receiver is 23 dBK, resulting in a G/T of 13.14 dB/K—higher than that reported in [29,30]. The classification of various beam steering techniques is illustrated in Figure 2.

**Figure 3:**
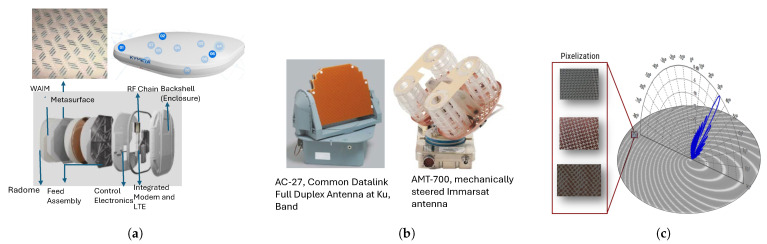
Commercially available antennas for SOTM and COTM applications. (**a**) Electronically Steered Antenna by Kymeta [43,44], (**b**) Mechanically Steered Antennas by Honeywell [45]. (**c**) Metasurface Antenna by Waveup [46].

Conventional beam-steering antenna technologies, such as electronically scanned phased arrays and mechanically scanned parabolic dishes, are well-established and widely deployed in various commercial applications. In contrast, passive metasurface-based beam-steering antennas are relatively new and are still being investigated to explore their full potential and capabilities. A high-level comparison of various beam steering techniques is given in Table 1, while some example designs for each category are illustrated in Figure 3.

**Table 1:** Performance comparison of different beam steering techniques.

Technique	Reconfigurable	Steering Mechanism	Feed Network Complexity	Metasurface Type	$\eta_{\mathrm{ap}}\left(\%\right)$	EIRP	G/T
Transmitarrays [32,33,34,35,36,37]	No	Mechanical Translation/rotation of feed or surface	Less	Passive	22.3–77.5	Medium to High	Medium to High
Reconfigurable Reflectarrays [38,39,40,41]	Possible	Electrical using PIN or Varactor diodes, and Motor in some cases	More	Active	7–69.41	Low to High	Low to High
Phased Arrays [29,42,43]	Possible	Electrical	More	Active	High	High	High
Near Field Meta-steering [31]	No	Mechanical rotation of metasurface	Least	Passive	Medium	N/A	N/A

$\eta_{ap}$: Aperture Efficiency, EIRP: Effective Isotropically Radiated Power, G/T: Gain to Noise temperature.

## 3. Scope and Methodology

Steering antenna systems have been classified in the literature based on various factors, including their size [47], the involvement of mechanical and non-mechanical movement [48], lens-based antennas supported by the European Space Agency [49], and other system properties [50]. Some previously reported articles focus solely on electronically steerable antennas, as seen in [44], while others discuss high-throughput SATCOM antennas, such as in [12]. Unlike earlier works on beam steering antennas, this article provides a comprehensive review of dual-band beam steering antennas that incorporate only passive metasurfaces. A passive metasurface-based antenna system can enable the design of high-gain, low-cost antennas with comparable G/T, thereby reducing overall antenna design complexity. The fundamental component in a passive metasurface-based beam steering system is the phase-gradient metasurface (PGM). This review also examines various unit cell topologies, which serve as the starting point for designing a dual-band PGM. The following key features are summarized in this article.

- The review encapsulates passive beam steering techniques focusing on full-duplex systems incorporating passive metasurfaces.
- Since the first step in designing a passive metasurface is finalizing the phase shifting cell, a subwavelength element that repeats periodically/aperiodically over the entire surface, careful design and selection are crucial and require deliberation. Therefore, the focus of this article is the design analysis of different dual-band phase transformation cell topologies available in the literature, with the pros and cons of each on the system-level parameters. This analysis aims to provide a valuable resource for designers beginning the development of dual-band PGMs used in various applications such as dual-band beam steering, dual-band phase correction, and dual-band lenses.

Given the emphasis on designing and developing dual-band PGMs, we briefly discuss the steering mechanisms that incorporate dual-band PGMs to provide context. These steering mechanisms can be categorized into three main types: transmitarrays (TAs)-based beam steering, reflectarrays (RAs)-based beam steering, and near-field meta-steering (NFMS). A brief overview of these techniques is provided in the following subsequent section. The rest of the paper is organized as follows: Section 4 briefly overviews the steering technologies incorporating phase-gradient metasurfaces. Section 5 discusses various unit cell topologies. This is followed by an analysis of the challenges associated with dual-band unit cell design and a review of different approaches implemented to address these challenges in Section 6 and Section 7. Finally, the paper provides a summary and concludes with a comparison of existing passive dual-band metasurfaces an integeral part of passive beam steering antenna systems.

## 4. Dual-Band Passive Metasurface Based Beam Steering Techniques

The antenna systems utilizing metasurfaces or metamaterials discussed in the literature can be broadly classified as RAs, TAs, and NFMS. Dual-band beam steering with RAs has mostly been achieved by utilizing reconfigurable RAs or a combination of RAs and phased arrays. On the other hand, the other two techniques use strategically designed metasurfaces to achieve dual-band beam steering capability. Beam steering in TAs is implemented either by feed translation/rotation or by rotating the transmitarrays (metasurfaces). In the case of NFMS systems, two PGMs are independently rotated synchronously or asynchronously parallel to the antenna axis and placed in the near-field of the antenna to achieve beam steering. This section briefly describes these three popular techniques along with state-of-the-art antenna systems. The inherent challenges associated with each approach are also discussed subsequently.

### 4.1. Reflectarray-Based Beam Steering

Introduced initially in 1963 [51], RAs are a type of antenna that combines the attributes of reflector antennas with array antenna principles. It consists of a feed antenna illuminating a reflecting surface. The feed is usually a horn antenna placed at a focal distance from the reflecting surface. In RAs, planar metasurfaces are mostly used to emulate the behavior of a curved reflector. These metasurfaces comprise an array of scatterers that produce a predefined phase shift to compensate for the path lengths of a radiated wave from the feed placed at the focal length from the surface. As a result, the beam can be tilted in any direction based on the arrangement of the phase-shifting scatterers. A comparative analysis of passive mechanically reconfigurable single band RAs is given in [52]. Reconfigurable RAs that comprise a combination of active and passive metasurfaces are presented in [53]; however, beam steering is not explored. Dual-band beam steering has been made possible with reconfigurable RAs or a hybrid of active and passive RAs that can change the phase shift of cells to make adaptive beam steering possible. Such RAs are reported in [38,54,55,56,57].

### 4.2. Transmitarrays-Based Beam Steering

Recently, another class of antennas known as transmitarrays has gained significant research focus, enabling the design of highly directive antennas with beam steering capabilities. They incorporate passive metasurfaces with properties similar to those of a lens but are planar and fabricated using printed circuit board (PCB) technology. Compared to lens antennas, they are lightweight, conformable, and reconfigurable (in beam shape, direction, and polarization) [58]. Similarly to a RA, in TAs, a feed source is placed at the focal point of the metasurface. Dual-band beam steering TAs are reported in [32,33,59,60,61,62,63,64,65,66,67,68,69]. In most steering mechanisms, in-plane feed translation or feed rotation is used to steer the beam, requiring additional feed displacement arrangements [32,33,67,68]. In some TAs, the rotation of the dual-band metasurface is used [59]. In [32], an optimal approach for designing a dual-frequency band TA is presented, exhibiting a measured gain of 24 dBi at 20 GHz and 27 dBi at 30 GHz. The consistent beam direction in both frequency bands with steering confirms the full-duplex operation in the dual-frequency bands. In [68], the dual-band TA operating at Ka-band, with a gain of 28.3 dBi and 28.4 dBi at the two frequency bands of 20 GHz and 30 GHz, is presented. Another dual-band beam steering TA using planar metasurfaces is presented in [59]. The metasurface comprised the double square loop resonant elements in the multi-layer unit cell, providing a steering range of around $\pm 50^{\circ }$, with the peak gain of 19.8 dBi at 14 GHz and 16.7 dBi at 8 GHz, respectively. The concept of beam steering with independent phase control using a folded TA is presented in [60]. The unit cell provides independent phase control for the dual frequency bands utilizing polarization conversion from linear to different circular polarization (RHCP and LHCP). Several types of multilayer dielectric, composite, and metallic phase-shifting surfaces or metasurfaces have been reported in the literature with linear- and circular-polarization-compatible unit cells.

### 4.3. Near-Field Meta-Steering Systems

One disadvantage of a TA is its lateral height because the feed is placed at a specific distance equivalent to the focal length from the metasurfaces. Another technology known as near-field meta-steering (NFMS) is proposed in [23] to reduce the total lateral height of the antenna and to avoid feed displacement for steering the beam. The design presented in [23] is for single-frequency band operation. Several modifications of single band NFMS are also presented in [70,71,72,73]. In a later work, an NFMS system that supports full-duplex communication is presented in [31]. In NFMS, the metasurfaces are placed within the near-field of a high-gain antenna system. The rotation of these metasurfaces about their axis in parallel to the antenna axis enables beam steering.

### 4.4. A Comparative Analysis of Antenna Beam Steering Based on RAs, TAs and NFMS

**Figure 4:**
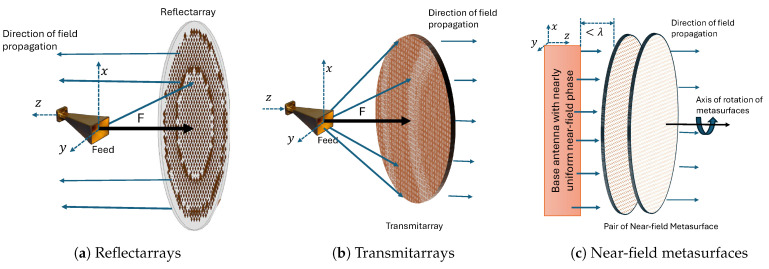
Dual-band metasurface based beam steering techniques.

Figure 4 illustrates the three different steering techniques discussed earlier in this section, showcasing the difference in steering methods based on their structure and operating principles.

In RAs, the reflection magnitude of the metasurface is always one (0 dB) due to the reflections of the entire incident wave from a metal ground plane; thus, only the reflection phase of the elements can be controlled. RAs also suffer from feed blockage issues [74]. Furthermore, most RAs have a bandwidth limitation of around 10% or less [74]. The feed must also be maintained at a specific distance from the reflecting surface to achieve better aperture illumination and higher gain. One advantage of RAs is that they can combine the positive features of both reflectors and phased arrays without needing additional phase shifters or power dividers [75].

The issue of feed blockage is resolved in TAs, as the metasurfaces are used in transmission mode, allowing planar beam steering antenna system designs for single-band, broadband, and dual-band applications with high gain while maintaining the pattern shape during beam steering. One disadvantage associated with TAs involving the feed-tuning technique is pattern degradation due to phase alteration during feed displacement. Another disadvantage is the requirement of placing the metasurface at a focal distance from the feed to maintain the F/D ratio, which notably hinders size reduction in TAs.

An advantage of NFMS systems over TAs is a significantly reduced overall system height since the metasurfaces are placed in the near-field region of the antenna. That said, dual-band high-gain NFMS systems are not as extensively explored in the literature compared to TAs. One future research direction could be to investigate dual-band and broadband NFMS systems for high-gain applications suitable for SOTM applications.

In the three steering mechanisms discussed in this section, the fundamental component of the system facilitating beam steering is a free-standing metasurface placed at various locations with respect to the feed, distinguishing the steering mechanisms from one another. In a RA, such a surface is used in reflection mode with a reflection coefficient close to 1 (0 dB), while in TA or NFMS systems, highly transmitting metasurfaces are used with a transmission coefficient close to 1 (0 dB). This review article further discusses various passive highly transmissive dual-band metasurfaces used in the literature to design the full-duplex beam steering antenna systems, i.e., TAs-based beam steering antennas and NFMS systems. These dual-band metasurfaces have been referred to as Phase Shifting Surface (PSS) [76,77,78], Phase-Gradient Metasurface (PGM) [24,79], time delay metasurface [23], discrete lens, planar lens, and flat lens [22,32] in the literature. In this article, these metasurfaces are consistently referred to as PGMs.

## 5. Passive Dual-Band Phase-Gradient Metasurfaces

Metamaterials are artificially engineered materials created using sub-wavelength elements, known as meta-atoms or unit cells [80]. Metasurfaces are the planar alternative to metamaterials and are sometimes referred to as 2D electromagnetic surfaces [81]. Metasurfaces are used for various applications, such as polarization conversion, filtering, designing phase-shifting surfaces and phase-gradient metasurfaces [22,24]. Passive PGMs are freestanding surfaces with a saw-tooth phase profile in a specific direction while maintaining a constant phase in the orthogonal direction. An array of distinct cells (periodically repeated in one of the directions, either *x* or *y*, while aperiodic in the orthogonal direction) determines this phase gradient, with each cell designed to provide a predetermined transmission phase shift to a normally incident electromagnetic wave. In some applications, the amplitude of the near-electric field can also be manipulated using these cells. We can refer to the antenna array theory to understand the working principle behind the PGMs. As per the antenna array theory [82], if several discrete identical radiating elements are arranged in a straight line, the field radiated by such an array can be given by Equation (1) in its simplest form.

(1) $$E_{\mathrm{array}}=E_{\mathrm{single}\;\mathrm{element}\;\mathrm{at}\;\mathrm{reference}\;\mathrm{point}}\times \mathrm{Array}\;\mathrm{factor}$$

where $E_{\mathrm{array}}$ represents the total electric field. The array factor depends on the number of elements (N), the geometrical properties of the elements, their spacing (d), their relative magnitudes and their relative phase. When all the array elements are identical, with equal magnitude and a progressive phase delay among the adjacent elements, the array is called a uniform linear array. For a uniform linear array, the array factor (AF) is given by Equation (2).

(2) $$AF\left(\theta \right)=\frac{\sin\left(\frac{Nkd}{2}\sin\theta \right)}{\sin\left(\frac{kd}{2}\sin\theta \right)},$$

where *d* is the spacing between the elements, *k* is the free space wave-number, and *N* is the total number of elements. The maxima of the beam occurs at kdsinθ=2mπ, where m=0,±1,±2,±3,…. Secondary maxima, known as the grating lobes, occur in array antennas when the inter-element spacing is large relative to the operating wavelength. These lobes occur at an angle away from the main beam, and their angular position can be calculated using Equation (3).

(3) $$\theta_{g}=\sin^{-1}\left(\frac{m\lambda }{d}\right)$$

The following condition must be satisfied to avoid the grating lobes in the visible region $\left(\theta =\pm 90^{\circ }\right)$ given in Equation (4).

(4) $$\frac{d}{\lambda }\leq 1-\frac{1}{N}$$

To direct the radiated beam away from the broadside direction, all the antenna elements are fed with a signal that has been phase-shifted. This is peformed so that the phase shift between adjacent antenna elements remains constant, denoted by $\phi_{step}$. For example, if we want to tilt the radiated beam at an angle $\theta_{o}$ away from the broadside direction, $\phi_{step}$ between adjacent elements can be set as $\phi_{step}=kd\sin\theta_{o}$. One method to achieve a constant phase shift among adjacent elements is directly exciting the feed with phase-shifted signals, often utilizing active phase shifters. Alternatively, the feed length (microstrip or strip-line length) can be adjusted so that each element receives a phase-shifted input.

**Figure 5:**
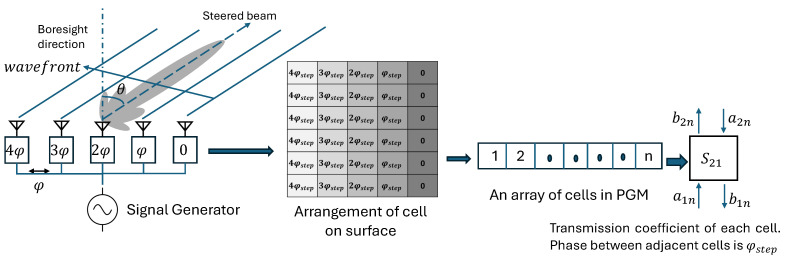
Analogy between an antenna array and array of cells arranged in a metasurface to exhibit a transmission phase gradient.

**Figure 6:**
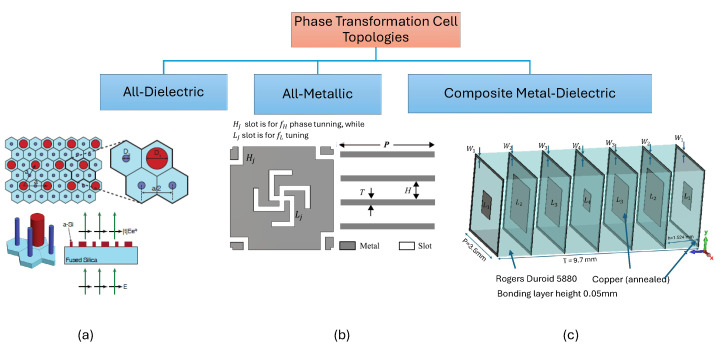
Different phase transformation cell topologies based on their implemented structure. (**a**) An all-dielectric multiwavelength cell [83], (**b**) a dual-band all metal cell [31], and (**c**) a dual-band composite cell redrawn based on the structure reported in [32].

Similarly to antenna arrays, in the case of passive PGMs, if an array of cells on the surface is arranged such that the phase difference between adjacent cells remains constant, denoted by $\phi_{step}$, the incident beam on the surface can be tilted to an angle $\theta_{o}$ away from the broadside direction, depending on this phase gradient. In Figure 5, we observe how an array of antenna elements with a phase difference from element to element equal to ϕ resembles a metasurface of cells with a gradient phase profile having an adjacent phase difference of $\phi_{step}$. The fundamental phase-shifting element in the PGM is a sub-wavelength cell. Based on structure and composition, the different types of cells available in the literature can be broadly classified, as shown in Figure 6.

**Figure 7:**
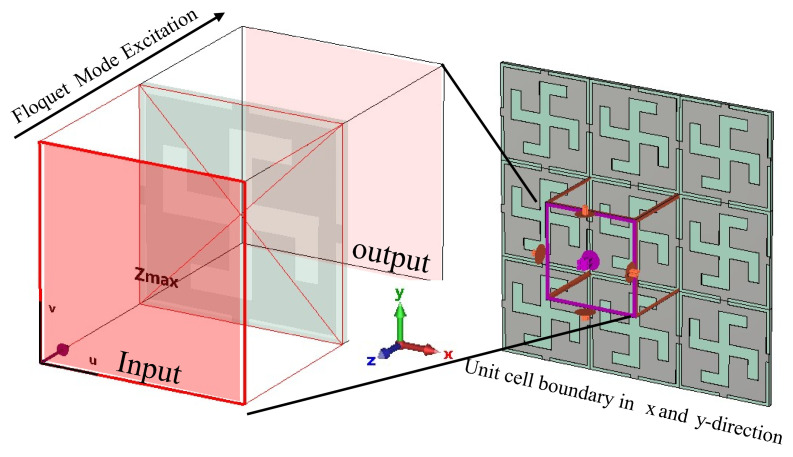
Unit cell simulations in CST, periodic in *x* and *y* axes, open in *z* axis with Floquet mode excitation.

**Figure 8:**
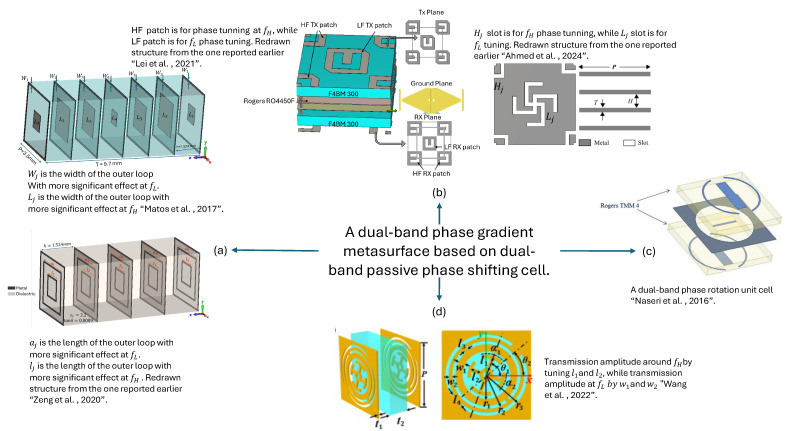
Different types of dual-band phase shifting cells. (**a**) Multilayer cells based on the selection of optimized phase pairs corresponding to the desired response in the dual-frequency bands [59,84], (**b**) interleaving resonant elements corresponding to each frequency [31,60], (**c**) dual-band phase rotation cell [61], and (**d**) a concentric cell using orthogonal polarized modified split rings and Jerusalem cross [67].

The first step in designing any metasurface is to find a suitable phase transformation cell topology. Since a phase-shifting cell is a fundamental element that is repeated with variations in its geometric properties on the surface, the overall performance of the antenna system greatly depends on the design and analysis of the cell. This phase shifting cell is initially simulated using periodic boundary conditions in the *x* and *y* directions while assuming Floquet mode excitation in the *z* direction to analyze its transmission coefficient phase and magnitude response, and hence is referred to as a unit cell when simulated with periodic boundary conditions. Floquet mode excitation in the CST- Microwave Studio (MWS) is shown in Figure 7. The different types of dual-band unit cells that have been used in the literature using the different structural topolgies are shown in Figure 8.

### 5.1. All-Dielectric Unit Cells

All-dielectric unit cells are explored in [85,86,87,88,89] for various beam steering and phase correction applications. Dual-frequency or multi-wavelength meta-molecules are presented in [83]. A hexagonal unit cell operating at two different frequencies and providing independent phase control is designed using four nano-posts placed at the center of the hexagonal cell. Three nanoposts are used to control the cell response at the higher frequency, while one nanopost is used to control the response at the lower frequency. The diameter of the nano-posts is varied to achieve phase variation, keeping their length constant. Using a holey substrate, a dual-band all-dielectric unit cell is also reported in [90] for dual-band phase correction in TAs. However, the structure may not be suitable for designing PGMs. A crucial obstacle exists in creating all-dielectric dual-frequency metasurfaces. While several designs necessitate more significant physical footprints, others involve complex machining operations, presenting substantial challenges in the manufacturing process. In addition, the design of all-dielectric unit cells to achieve dual-frequency band capabilities may raise problems regarding feasibility.

### 5.2. All-Metallic Unit Cells

Single band all-metallic unit cells are also explored in [70,73,90,91,92], eliminating the need for expensive laminates in all-dielectric or composite (metal-dielectric printed) structures. The first dual-band all metallic phase gradient near-field meta-steering antenna is reported in [31] utilizing dual-band all metallic unit cells incorporating a modified swastika slot in the middle for phase adjustment at one frequency band of operation and half swastika slots in the corners for the other frequency band’s phase tuning. Overall, four metal and dielectric layers separated by air gaps are utilized.

However, limitations of these structures include narrow operation bandwidth, losses due to the conductivity of metallic layers, expensive manufacturing, and polarization dependence. Moreover, most of these cells are fabricated by cutting slots in the metal sheets using the concentrated heat of high-power lasers. When subjected to high-intensity heat, the metal sheets may deform, which can be a significant obstacle in extending such designs for highly directive (>30 dBi) applications. Most of the all-metal structures are in the form of mesh, which may become very thin for higher frequencies, and bending of the surface may be another issue when using such unit cells.

### 5.3. Composite Metal-Dielectric Unit Cells

Composite (metal-dielectric) unit cells are made up of both metal and dielectric material. A detailed analysis of the choice of resonating element for such unit cells was presented in [93]. Multilayer printed unit cells can be used to design the metasurfaces. For implementing metasurface, only those cells that offer high transmission magnitude, ideally less than −1 dB, are selected. However, when the complete phase range is impossible using only −1 dB cells, a few cells with up to −3 dB transmission magnitude can also be selected. According to the detailed analysis of metasurfaces conducted in [94], the transmission phase of any single layer (one conductor layer placed over a dielectric substrate) surface is a function of substrate electrical thickness $\beta h_{d}$, where β is the phase constant ($\beta =\frac{2\pi \sqrt{\epsilon_{r}}}{\lambda_{0}}$) and $h_{d}$ is the height of the substrate. With a single-layer unit cell, the maximum phase range that can be achieved regardless of the implemented metallic element shape is $54^{\circ }$ with a transmission coefficient magnitude within −1 dB and $90^{\circ }$ with a transmission coefficient magnitude greater than −3 dB. To increase the phase range, the number of layers must be increased.

Wide-band dual resonant double square ring unit cells are presented in [95]. The unit cell structure has four metal-dielectric layers separated by an air gap. Adding the inner rings increases the achievable phase range and improves the 1 dB gain bandwidth by 7.5%. For any selected unit cell geometry, the phase range of a multi-layer unit cell structure depends on the substrate material, the number of layers, and the spacing between the layers. A minimum three-layer structure with two dielectric layers and three metal layers is required to provide a phase range of $360^{\circ }$, also at the cost of a reduced transmission coefficient magnitude of −3 dB. Increasing the number of layers increases the phase range with a transmission coefficient magnitude closer to 0 and more significant than −1 dB. It is also deduced that the electrical thickness of a substrate $\beta h_{d}$ should be at least 90 degrees at the resonant frequency to achieve the maximum transmission phase range. The complete analysis of two-, three-, and four-layer FSSs is given for different relative permittivity values inimproves [94]. It is concluded that the height of the dielectric layer should be selected according to the required phase range and the dielectric permittivity. If $\beta h_{d}$ decreases below 90 degrees, the phase range is reduced to 120 degrees. If $\beta h_{d}$ increases above 90 degrees, the transmission phase range is reduced to 240 degrees. For a three-layer FSS, with an increase in relative permittivity, the phase range increases. For $\epsilon_{r}=4.7$ and $|S_{21}|\geq -3$ dB, a transmission phase range of 360 degrees can be achieved with a four-layer unit cell with a reasonable transmission magnitude of $|S_{21}|\geq -1$ dB. The analysis included conducting experiments with different shapes of unit elements.

The main challenge in building a thin and lightweight metasurface is to achieve a complete 360-degree phase range while minimizing the number of layers. This optimization is essential to reduce the design expense. To design a dual-band beam steering system, the unit cell should be able to resonate in two different frequency bands. Many structures reported in the literature focus solely on the dual-band metasurface design without addressing the beam steering aspect. Designing a dual-band unit cell for a dual-band phase gradient metasurface is more challenging than designing a single-band PGM, primarily due to the need for independent $360^{\circ }$ phase range coverage required at the dual frequency bands, which is often achieved by varying the dimensions/rotation angle of the resonant elements of the cell.

Most cells reported in the literature are selected based on identifying phase pairs that simultaneously provide the required phase for both frequencies while maintaining a high transmission magnitude. However, this process is time-consuming, involving rigorous parameter sweep simulations, in which cell parameters are varied to analyze the phase response at both frequencies. This generates data sets with the maximum possible phase combinations for two frequencies with a high transmission coefficient magnitude. The highly transmitting cells are then selected from these generated data sets depending on the required phase profile of the metasurface. Such dual-band PGMs are reported in [32,61,69,96,97], where the phase shift through the cell is tuned either by varying the length or width of the geometric shape within the cell or by rotating the shape itself at various angles within the cell. In [32], phase tuning is achieved using phase delay (PD) unit cells, and a generic methodology to reduce the complexity of dual-band metasurface is presented. This work is an extension of the earlier work in [84,98]. According to [32], the cells corresponding to the frequencies $f_{1}$ and $f_{2}$ can be designed using square loops corresponding to the lower frequency and square patches corresponding to the higher frequency. Ref. [32] further suggested that if the frequency in the two bands is selected to maximize the greatest common divider between them, it will result in fewer non-repeated cells to achieve $360^{\circ }$ independent phase wrapping at the dual frequency bands. In the given analysis, the cell period is λ/4×λ/4 for the lower-frequency band and λ/3×λ/3 at the higher-frequency band. The reported unit cell is a seven-layer structure, and cell sizes are optimized to obtain the specific dimensions, giving required phases with high transmission coefficient magnitudes for both bands. Along with the time-consuming cell selection process discussed previously, another design limitation is utilizing seven metal and six dielectric layers, leading to a thicker metasurface. These layers are also bonded together, increasing the overall cost and complexity of the design and fabrication process. A comparison of the response of PD and phase rotation (PR) unit cells in terms of the scattering parameters of the surface is also discussed in [99]. The paper compares the working principles of PD and PR cells, their effect on the polarization of incident waves, and their design complexity.

In another study, a dual-band cell is introduced with circular polarization, operating at frequencies of 20 GHz and 30 GHz [68]. The unit cells designed for each frequency allowed independent phase variation at the respective bands, achieved by adjusting distinct cell parameters separately. To achieve this, the resonating element for the higher frequency band is strategically positioned at the corners of each cell. This placement ensures that when the cells are arranged in a periodic lattice, they formed complete resonating elements. In contrast, the resonating element is placed in the center of each cell for the lower-frequency band. This approach of interleaving the resonating elements specific to different frequency bands reduced the computational time required compared to previous techniques that aimed to identify common phase pairs. The overall structure of the proposed design consists of three layers, comprising three metal and three dielectric layers, with dual-polarized slot elements. Beam steering is achieved by translational sliding of the TA relative to the feeder. The proposed unit cell design is also converted to an all-metallic structure, eliminating the requirement of expensive laminates and making the cell suitable for high-temperature applications. The same approach of strategically positioning the resonant elements within the unit cell to achieve independent frequency tuning at the dual frequency bands is also utilized in [33,60,63]. Interleaved cells, however, acquire more space. Arranging the elements in a gradient metasurface while avoiding corner discontinuities can also be challenging.

A linearly polarized unit cell with vertical and horizontal dipoles corresponding to the dual frequency bands is also utilized to design a dual-band TA for wide beam scanning in [33]. The proposed design can be used for the uplink and downlink Ka-bands. Beam scanning of $\pm 40^{\circ }$ and $\pm 30^{\circ }$ is achieved independently at the two frequency bands. The results suggested that the transmitted beam can be scanned over $80^{\circ }$ for 19.5 GHz and $65^{\circ }$ for 29 GHz in elevation with only 2 dB scan loss in the peak gain. Displacement of the feed horn antenna at the focal distance from the surface is proposed for tilting the beam. Another similar dual-band unit cell with double horizontal and vertical dipoles was reported in [64].

Another dual-band design allowing independent phase tuning through the cell at dual frequency bands is reported in [67]. The reported cell is a single-layered substrate meta-atom. The two metallic layers at the top and bottom of the substrate layer are composed of a modified Jerusalem cross-resonator seated in a circular hole, a modified complementary split-ring resonator, and a ring connector. The modified Jerusalem cross resonator is used to tailor the phase of the cell at the lower-frequency band $f_{L}$. In contrast, the modified complementary split-ring resonator is utilized to tailor the phase at the higher frequency band $f_{H}$. In this case, the PR technique independently tunes the phase response at the dual frequency bands. In [59], a dual-band lens is implemented, operating in orthogonal polarization for the X-band and Ku-band. A rectangular dual-band cell with two orthogonal rectangular patches printed on four metal layers separated by three dielectric layers is used. At $f_{L}$ = 8 GHz, a y-polarized feed is used, with the resonant elements’ dimensions parallel to the y-direction tuned for phase control. At $f_{H}$ = 14 GHz, an x-polarized feed was used, with the resonant elements’ dimensions parallel to the x-axis being tuned for phase adjustment.

Recently, another dual-band cell with independent frequency tuning has been reported in [100]. The unit cell consisted of four metal layers and four dielectric layers of a thin substrate. It is composed of interleaved square slot and cross slot resonant elements, following the orthogonal principle of resonance to allow independent phase tuning at the dual frequency bands.

The composite unit cells reported in this section provide more design flexibility and wider bandwidth at the dual frequency bands of operation. Moreover, such PGMs can be precisely fabricated at higher-frequency bands using the standard printed circuit board fabrication techniques. However, high-performance laminates are costly. The dielectric electric field breakdown threshold is also lower than that of metals, making it less feasible for space applications or where environmental conditions are harsh [31,100].

Based on the structural topology of a unit cell, the performance of different cell can be summarized as in Table 2.

## 6. A Comparison of Different Dual-Band Phase-Transformation Cells

**Figure 9:**
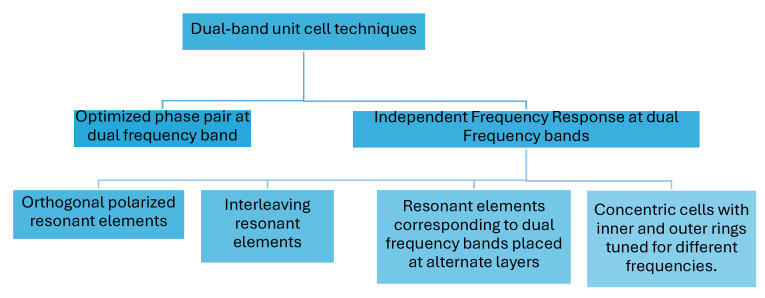
Summary of techniques to design a dual-band unit cell for passive phase-gradient metasurfaces.

Figure 9 summarizes the techniques previously used to design a dual-band PGMs incorporated into a TAs or NFMS to achieve beam steering.

Based on the literature review presented above, a comparison of different relevant unit cell topologies used for phase correction or beam steering is presented in Table 3. The unit cell topologies investigated in this work extend beyond those specified in Table 3. However, our focus remains on those relevant to dual-band passive PGM design. The survey reveals that the independent performance of the unit cell in each frequency band provides greater flexibility in the design of TAs or NFMS for full-duplex steering applications. When the unit cell’s response is independent at the two frequency bands, it allows for arbitrary beam direction. Coincident beam direction at the dual frequency bands is preferred in full-duplex communication systems. In such cases, a cell with an independent phase response can help avoid tedious and time-consuming phase optimization techniques. Dual-polarized cells are also preferred over linear-only polarization since they can accommodate any polarization, circular, linear, or slant.

The summary presented in Table 3 shows that achieving an independent phase response across a broader frequency range is a relatively underexplored area. Many dual-band cell topologies rely on optimizing phase pairs at specific frequency bands, necessitating a lengthy design process. Moreover, when the frequency separation is large, the interleaved placement of resonant elements at the dual frequency bands results in larger cells at the higher frequency band. Grating lobes appear for different wave-incidence angles if the cell size exceeds half the wavelength.

## 7. A Comparative Analysis of Various Reported Dual-Band Passive Metasurfaces Based Antenna Systems

The key performance metrics achieved by state-of-the-art full-duplex antenna systems based on passive dual-band metasurfaces are summarized in Table 4.

Based on the data reported in various works in the literature, it can be summarised that NFMS offers the most compact solutions in terms of antenna height, easier to fabricate, and lighter in weight. On the other hand, the aperture efficiency of a TA or RA is greater than that of the NFMS. A summary of this comparison conducted for different dual-band beam steering systems incorporating PGMs is given in Table 5.

## 8. Conclusions

Beam steering is essential in the modern communication landscape dominated by technologies like SATCOM and COTM. Mass-produced, cost-effective solutions are necessary to meet the expanding consumer base. Beam steering antennas based on passive planar metasurfaces offer compact, low-profile, and cost-effective solutions with steering capabilities similar to phased arrays but without the need for active RF components that require heat sinks and complex feed networks. These planar metasurfaces, specifically known as phase-gradient metasurfaces, enable precise control over the direction of electromagnetic waves by incorporating passive phase-shifting/phase-transforming cells. This article presents a comprehensive analysis of various design techniques found in the literature for dual-band phase-shifting cells in PGMs. These designs aim to enable full duplex communication using a single shared aperture.

Since the fundamental element in a PGM is a phase-transforming cell, selecting a suitable dual-band unit element is crucial to ensure the desired performance of the overall steering antenna system. The dual-band cells described in the literature have been comprehensively classified based on their structure in this article. Furthermore, to achieve dual-band resonance, five main techniques have been utilized in the reported designs for the selection and tuning of resonant shapes within the cells.

- Optimized phase pair [32,59,84]: This technique may result in the design of concentric cells and smaller unit cells but requires more optimization and complex computations with larger datasets.
- Interleaved resonant elements [31,33,60,63,64,65,66,68,69,83,100,101]: This technique enables independent tuning but may result in larger cell sizes that may lead to phase quantization error or grating lobes particularly at a higher-frequency band. The placement of cells to form the PGM needs careful investigation to avoid corner element shape discontinuities. Smaller unit cell topologies need to be explored to design a PGM that can allow better resolution in phase correction/tunning and enhance the overall steering range of the antenna system.
- Orthogonal polarised resonant elements [61,62,63,64,100]: Such cells allow independent phase tuning at the dual frequency bands, minimising the need for time-consuming computations. Unit cell miniaturization may be further explored to improve phase quantization errors.
- Layer separation [61,102]: This approach may lead to an increased number of layers.
- Concentric cell [67]: The designed cell has a modified Jerusalem cross resonator and a modified complementary split ring resonator to tune transmission properties at two frequencies independently. The overall cell is concentric but at the cost of complex geometry for the overall cell.

Nevertheless, each technique has its own advantages and disadvantages, necessitating careful consideration in the metasurface design. From analyzing various unit cell topologies, several research gaps have been identified.

All-metallic phase-shifting cells, suitable for space applications, allow operation under high power without requiring expensive laminates. However, these structures suffer from narrow bandwidth. Exploring waveguide-based dual-band all-metallic phase-shifting cells could offer higher bandwidth along with improved structural rigidity.

Composite metal-dielectric phase-shifting unit cells can provide an improved bandwidth response, but most of these composite cells require multiple layers of metal and dielectric material to achieve maximum phase range at dual frequency bands, increasing the overall thickness of the structure. Dual-band subwavelength cells with fewer metal-dielectric layers that can offer a better phase range while reducing the number of alternating layers can be further explored. The incident field for the majority of feed antennas is concentrated at the center, so high transmission magnitude cells can be placed in the center, while low transmission magnitude cells with desired phase shifts can be positioned towards the periphery. This approach of amplitude tapering, coupled with height reduction, can be explored to achieve better performance. Moreover, most dual-band cells have been optimized for normal angles of incidence. The stability of a unit cell’s response under oblique angles of incidence at the two frequency bands needs further investigation. The various planar cell topologies discussed in this review can be easily integrated into existing RF front-end systems for SATCOM and COTM platforms. The choice of material for implementation depends significantly on the nature of the application. As an example, all-metallic structures are well-suited for space applications due to their ability to withstand extreme temperatures and harsh conditions. In contrast, composite metal-dielectric materials are more appropriate for applications requiring wider bandwidth and commercial COTM operations. Another hurdle associated with all-metal metasurfaces is that, since these panels are manufactured using laser cutting, high-frequency structures may require a level of precision that may not be feasible with the existing technologies. In such cases, printed structures could be preferred for high-frequency operations. However, low-loss laminates may be expensive, which could be a limitation for the large-scale production of printed metasurfaces.

Although several variations of passive metasurfaces have been reported, reconfigurability, as seen in active reflectarrays, has not been extensively explored. Further research into reconfigurable metasurfaces is needed to dynamically control the beam direction. Moreover, the miniaturization of cells, while maintaining independent dual frequency response and achieving a wider bandwidth in dual frequency bands, remains a significant challenge for designing shared aperture antennas based on passive metasurfaces that can provide compact and cost-effective solutions with lower power consumption for the future communication systems catering to the ever increasing demands for global connectivity.

All the technologies discussed in the article are essentially state-of-the-art and have undergone constant evolution in the short time since their inception. While a one-on-one comparison might not be technically correct, the paper aims to present their working mechanisms, with the choice of technology ultimately depending on specific design requirements. Technologies like phased arrays, reconfigurable intelligent surfaces, TAs, and RAs have gained significant interest. Many have found their way into industrial applications, with some being readily used in the industry, while some techniques like shared aperture beam steering TAs, and low-profile, and cost-effective shared aperture NFMS are still under extensive research with, their full potential yet to be explored.

## Figures and Tables

**Table 2:** Summary of performance of different materials used for cell design.

Cell Topology	Dual-Band Design	Op. Freq. BW	Fabrication, Cost	High Temp. App.
All-Dielectric	Complex	High	Cheaper if using PLA or ABS	Lose structural integrity
All-Metallic	Easy	Low	Laser cutting, suited for flat panels only	Suitable
Comp. Metal-Dielectric	Easy	Medium	Traditional printing technique, require expensive laminates	Suffer dielectric breakdown

Op. Freq. BW: Operational Frequency Bandwidth, Temp.: Temperature, App.: Application, Comp.: Composite.

**Table 3:** Specifications of resonant elements in various references.

Ref.	Resonant Element	Topology	Number of Layers	Cell Size	Frequency Capability	Polarization	Phase Control
[95]	Double Square Loop	Composite separated by air gap	4	0.6λ	Wideband (29–32 GHz)	CP	NA
[101]	4-arm structure for one band, 4-leg structure for other-band	Composite	One metal and one dielectric layer	$0.2\lambda_{L}$	Dual-band Ku/Ka (14.1–16 GHz and 29.2–36.8 GHz)	Dual	Separate tuning of elements by length variation
[102]	Cross dipole for higher frequency, backed by square loops for lower frequency	Composite interleaved structure	Five dielectric and seven metal layers	$0.3\lambda_{L}$ and $0.4\lambda_{H}$	Dual-band (20, 30 GHz)	CP	NA
[69]	Split ring center elements for lower frequency, quarter split circle at corners for higher frequency	Composite	Five metal and four dielectric layers	0.53$\lambda_{L}$ and 0.8$\lambda_{H}$	Dual-band (20, 30 GHz)	CP	Independent phase tuning by rotation of split rings
[84]	Square Patch Middle and square loop mentions shape against design frequ	Composite	seven metal and six dielectric layers	$0.35\lambda_{H}$	Dual-band (20, 30 GHz)	CP	Optimal Phase Pair Selection
[60]	High frequency and low frequency patches	Composite	Three metal & two dielectric layers, bonded together	0.49$\lambda_{L}$ and 0.6$\lambda_{H}$	Dual-band (12, 15 GHz)	Dual CP	NA
[68]	Swastika cross slot for lower frequency, half cross slot on cell corners for higher frequency	Composite, Thin substrate separated by air gap	Three metal and three dielectric layers	$0.53\lambda_{H}$	Dual-band (20, 30 GHz)	CP	Independent Frequency tuning by length variation
[59]	Three concentric square loops	Composite structure, bonded layers	Five metal & four dielectric layers	$0.38\lambda_{H}$	Dual-band (8, 14 GHz)	CP	Optimum Phase pair by length variation
[33]	Vertical and Horizontal Dipoles	Composite Bonded Layers	Three metal and three dielectric layers	$0.58\lambda_{H}$	Dual-band (20, 30 GHz)	LP	Independent frequency tuning as the resonating elements are cross-polarized
[65]	Cross slot and magnetic dipole slot	All metal	Three metal layers	$0.33\lambda_{H}$	Dual-band (11, 12.5 GHz)	CP	Independent frequency tuning as the resonating elements are cross-polarized
[31]	Modified swastika slot in the middle for LF, and half swastika slot in corners for HF	All metal, separated by air gap	Four metal layers	$0.5\lambda_{H}$	Dual-band (Ku)	CP	Optimum phase pair with partially independent phase response

**Table 4:** Key performance comparison of dual-band beam steering antennas incorporating passive metasurfaces.

Ref.	Steering Mechanism	Operating Frequency (GHz)/Bandwidth (%)	Steering Range (°)	Total Antenna Height	Peak Gain (dBi)	Gain Variation/Scan Loss (dB)	Technique
[59]	Metasurface Rotation	8, 14/N-A	±52.7,±49.5	$\;5\lambda_{H}$	16.7, 19.8	1.7	TA
[31]	Metasurface Rotation	11.9, 14.2/3.4, 7.2	±46,±51	$2.5\lambda_{H}$	15, 19.8	3.0	NFMS
[60]	Patch Rotation	12, 15/8.8, 9.1	±45	$1.7\lambda_{H}$	25.3, 24.9	N-A	TA
[67]	Feed Translation	20, 30/10, 6.7	±60	$2.8\lambda_{H}$	18, 17.7	3.9, 3.5	Metalens
[68]	Feed Translation	20, 30/10, 7	±30	$\approx 6\lambda_{H}$	15.3, 15.5	2.3, 3.6	TA
[59]	Metasurface Rotation	X, Ku/N-A	±52	$11.2\lambda_{H}$	16.7, 19.8	5.4, 5.3	TA
[32]	Feed Translation	20, 30/N-A	±50	$12\lambda_{H}$	29, 27	3.6, 3.3	TA
[103]	Feed Translation	20, 30/N-A	+52.5,+51.6	≥6.24$\lambda_{H}$	21.8, 24.3	2.6, 2.7	TA
[33]	Feed Translation	19.5, 29/10.8, 11.7	±40,±35	≥11$\lambda_{H}$	30, 27	2.0	TA
[35]	Feed Translation	19.8, 28.2/19.8, 12.0	±20,±30	$\approx 11\lambda_{H}$	27.1, 29.9	1.5, 3.4	TA
[36]	Feed Translation	3.5, 28/N-A	112,56		6.4, 19.6		Multiple Antenna
[66]	Feed Translation	19.5, 29/11.3, 11.4	±20,±18	$10.7\lambda_{H}$	25.9, 29	3.0, 3.2	TA
[62]	Translation and rotation	20.4, 29.6/4, 8	±50,±50	$12.4\lambda_{H}$	23.4, 25.3	1.8, 2.5	TA

N-A: Not Available, TA: Transmitarray, NFMS: Near Field Meta-steering.

**Table 5:** Summary of analysis for dual-band beam steering antennas incorporating metasurfaces.

Parameter	NFMS	TA	RA
Feed Blockage	No	No	Yes
Antenna Overall Height	Least	Higher	Higher
Aperture Efficiency	Medium	Higher	Higher
Dual-band beam steering	Easier	Easier	Complex

## Data Availability

Data are contained within the article.

## Abbreviations

The following abbreviations are used in this manuscript:

SATCOM	Satellite Communications
COTM	Communication on the move
ITU	International Telecommunication Union
LEO	Lower Earth Orbit
MEO	Medium Earth Orbit
GEO	Geostationary Earth Orbit
EIRP	Effective Isotropically Radiated Power
G/T	Gain-to-noise temperature
PGM	Phase-Gradient Metasurface
RF	Radio Frequency
SOTM	SATCOM on the Move
NFMS	Near-Field Meta-Steering
RA	Reflectarray
TA	Transmitarray
RHCP	Right Hand Circularly Polarized
LHCP	Left Hand Circularly Polarized
PSS	Phase Shifting Surfaces
MWS	Microwave Studio
PD	Phase Delay
PCB	Printed Circuit Board
PR	Phase Rotation
